# Heart rate variability and its modulation by nutrients: a narrative review on implications for cardiovascular aging

**DOI:** 10.3389/fnins.2025.1654796

**Published:** 2025-12-08

**Authors:** Youyou Zhao, Peng Chen, Yongxin Zhang, Shaofeng Huo, Danyang Yu, Xinyi Zeng, Weiguo Zhang

**Affiliations:** 1 R&D, Sirio Life Technology (Shanghai) Co., Ltd., Shanghai, China; 2 R&D, Sirio Pharma Co., Ltd., Shantou, Guangdong, China; 3 Zyxel Inc., Carrollton, TX, United States; 4 Las Colinas Institutes, Irving, TX, United States

**Keywords:** intercellular and interorgan communication, resting heart rate (RHR), heart rate variability (HRV), cardiovascular aging, inflammation, longevity, nutrients

## Abstract

Altered intercellular communication is a hallmark of aging, influencing systemic processes across the cardiovascular, neurological, and psychological systems. Among these, cardiovascular aging is particularly important due to its strong association with morbidity and mortality in industrialized societies. Heart rate variability (HRV) reflects the natural fluctuations in the time intervals between heartbeats and serves as an indicator of neural interorgan communication, particularly within the cardiac and neural systems, which is fundamentally underpinned by intercellular signaling. HRV captures the autonomic nervous system’s regulation of cardiac function, reflecting the balance between sympathetic and parasympathetic activity. Beyond its mechanistic significance, HRV provides a window into the organism’s adaptive capacity to stress and the maintenance of homeostasis, encapsulating the interplay between cardiovascular, neural, and endocrine systems. Increasing evidence recognizes HRV as a predictor of survival across diverse populations, from patients with cardiovascular, metabolic, and neurological disorders to the general population. Importantly, HRV is modifiable, making it a compelling target for interventions aimed at enhancing healthspan and lifespan. Among these interventions, nutritional strategies hold particular promise. This review synthesizes current evidence on the impact of key dietary factors including omega-3 fatty acids, vitamin B12, and calorie restriction on HRV modulation in human studies. These findings underscore the potential of nutritional approaches to mitigate the deleterious effects of cardiac and neural aging while promoting systemic resilience. HRV transcends its role as a biomarker, serving both as an independent outcome measure and a critical component of algorithmic models for evaluating the efficacy of aging-related interventions. By advancing our understanding of HRV and its modulation through diet, this review bridges fundamental aging biology with applied clinical strategies, highlighting its transformative potential in optimizing both quality of life and longevity.

## 1 Introduction

Intercellular and autonomic communication is essential for coordinating biological functions in multicellular organisms (Alberts et al., 2022). At the cellular level, intercellular communication occurs via direct cell-to-cell contact or through chemical signals, including autocrine, paracrine, endocrine, and neural pathways, enabling cells to sense and respond to internal and external cues to maintain local and systemic homeostasis (Lopez-Otin et al., 2023). At a higher organizational level, these same mechanisms underpin interorgan communication, whereby different organs and tissues coordinate their functions to preserve organismal homeostasis. Dysfunction in either intercellular or interorgan communication contributes to the pathogenesis of many chronic diseases, including neurodegenerative and cardiovascular disorders, which often develop in a time-dependent manner with aging (Hanahan and Weinberg, 2011; Mattson and Arumugam, 2018; Abdellatif et al., 2023; Arakaki et al., 2023). Mechanistically, aging is associated with progressive alterations in neural, neuroendocrine, and hormonal signaling pathways including the adrenergic, cholinergic, dopaminergic, insulin/IGF1, gonadal, and immune systems, which compromise both intercellular fidelity and interorgan coordination (Deak and Sonntag, 2012; Santulli and Iaccarino, 2013; Gamage et al., 2020; Hagg and Jylhava, 2021; Abdellatif et al., 2023).

In the human body, the cardiovascular system is particularly sensitive to alterations in intercellular communication. Heart rate and rhythm are key indicators of cardiovascular health and disease (Massin et al., 2000; Lahiri et al., 2008), reflecting the frequency and regularity of cardiac cycles (Shaffer and Ginsberg, 2017). These measurable parameters provide critical insights into circulatory health and predict morbidity and mortality (Lombardi, 2000; Zhang and Zhang, 2009; Palatini, 2021). Elevated resting heart rate (RHR) has been considered an emerging risk factor for both cardiovascular and all-cause mortality (Cook et al., 2006; Zhang and Zhang, 2009; Zhang et al., 2016; Aune et al., 2017). Together with blood pressure, RHR contributes to the establishment of organ-specific clock models for biological age assessment (Tian et al., 2023; Fong et al., 2024).

While RHR is a straightforward and valuable measure of cardiovascular health and disease risk, it may not fully capture the complexity of the cardiovascular control or its regulatory mechanisms (Zhang et al., 2024b). Unlike a metronome’s steady rhythm, the heart exhibits subtle variations in the intervals between consecutive beats—a phenomenon known in cardiac physiology as heart rate variability (HRV). Primarily, HRV reflects the dynamic interplay between parasympathetic (primarily vagal) and sympathetic fibers of the autonomic nervous system (ANS) (Billman, 2013). Beat-to-beat fluctuations are modulated by baroreceptor reflex (a negative feedback system that maintains blood pressure within a normal range by adjusting heart rate and blood vessel constriction) and chemoreceptor reflex (a negative feedback mechanism that regulates ventilatory drive to maintain arterial pressures of oxygen [O_2_] and carbon dioxide [CO_2_] and pH within a narrow range), which continuously sense changes in blood pressure and blood chemistry, triggering reflexive adjustments via autonomic pathways to maintain cardiovascular homeostasis (Karim et al., 2023). High HRV indicates stronger vagal activity and balanced sympathetic output, reflecting well-coordinated intercellular and neural communication whereas declining HRV is generally considered a hallmark of aging and an independent predictor of adverse cardiovascular events and mortality (Jandackova et al., 2016; Sessa et al., 2018).

Aging is characterized by systemic challenges such as chronic low-grade inflammation—commonly referred to as *inflammaging* (Giunta et al., 2024), as well as increased oxidative stress (Mehdi et al., 2021), and the gradual functional decline of the cardiac and ANS. Although HRV typically declines with age, this inverse relationship is not linear; in advanced age, higher or stable HRV values may reflect rhythm irregularity rather than preserved autonomic flexibility. Consequently, HRV interpretation in older adults requires caution, particularly when based on short-term recordings. Additionally, abnormal cardiac electrophysiology such as atrial fibrillation or premature ventricular contractions (PVCs) can confound HRV measurements, where elevated HRV may represent a spurious effect rather than healthy aging. Nonetheless, the ANS remains responsive to modifiable factors, and accumulating evidence demonstrates that nutritional and lifestyle interventions can favorably influence HRV and autonomic function (Young and Benton, 2018; Lopresti, 2020; Struven et al., 2021). Specific macro- and micronutrients directly intersect with these age-associated pathways, offering a critical, yet often under-examined, mechanism through which the deterioration of HRV may be slowed or mitigated.

For instance, dietary patterns rich in anti-inflammatory and antioxidant compounds can directly support endothelial function and dampen the systemic inflammation that drives ANS dysregulation (Dong et al., 2011; Zhang and Tsao, 2016; Bruno and Ghiadoni, 2018). By influencing the metabolic environment and modulating intercellular signaling, nutritional components can potentially bolster the activity of the parasympathetic nervous system—the primary driver of high HRV thereby enhancing cardiac adaptability and resilience in older individuals. This focus on nutritional modification as a targeted strategy to preserve HRV in the context of aging represents the main distinguishing characteristic of this review, moving beyond general cardiovascular health to specifically address autonomic reserve.

This narrative review summarizes representative studies linking autonomic regulation, HRV, and systemic aging, emphasizing the modulatory influence of nutritional factors and the physiological mechanisms through which they sustain autonomic and cardiovascular health.

## 2 HRV: a marker of autonomic regulation and systemic aging

As previously noted, HRV quantifies the fluctuations in time intervals between consecutive heartbeats (Zhang et al., 2024b). Traditionally, HRV has been derived from electrical signals such as electrocardiograms (ECG) or 24-h Holter recordings, which provide high-fidelity data but are often limited by discomfort and restricted mobility associated with wearing wired devices. In recent years, HRV has also been obtained from mechanical waveforms such as pulse rate and blood pressure, or from photoplethysmographic signals (e.g., peripheral oximetry) (Schafer and Vagedes, 2013; Ernst, 2017; Task Force of the European Society of Cardiology and the North American Society of Pacing and Electrophysiology, 1996). With the advent of portable technologies including smartwatches, rings, and other wearable sensors, HRV measurement has become more accessible (see later discussion).

### 2.1 HRV analysis techniques

HRV can be assessed using several analytical approaches that capture distinct but complementary aspects of autonomic regulation. The three principal classes are time-domain, frequency-domain, and nonlinear analyses, each reflecting different dimensions of heart rate dynamics.

#### 2.1.1 Time-domain analysis

This method quantifies beat-to-beat fluctuations (R–R intervals) using statistical indices derived directly from ECG recordings. Common parameters include the standard deviation of normal-to-normal intervals (SDNN) reflecting overall HRV and combined sympathetic–parasympathetic influences, and the root mean square of successive differences (RMSSD), which specifically indexes parasympathetic activity. These straightforward metrics provide an accessible and reliable means of evaluating autonomic adaptability to internal and external stressors.

#### 2.1.2 Spectrum (frequency-domain) analysis

To further elucidate autonomic dynamics, HRV signals can be decomposed into frequency components using Fast Fourier Transform (FFT) or autoregressive (AR) modeling. The AR method, in particular, produces smoother spectral estimates and greater resolution for short data segments, making it widely used in HRV research. Spectral analysis separates HRV into three main bands: high frequency (HF, 0.15–0.4 Hz), low frequency (LF, 0.04–0.15 Hz), and very low frequency (VLF, <0.04 Hz). Traditionally, HF power is considered a marker of parasympathetic activity, whereas LF power reflects a mixture of sympathetic and parasympathetic modulation. Based on these interpretations, the LF/HF ratio has been widely used as an index of “sympathovagal balance,” with higher values assumed to indicate a shift toward sympathetic dominance and lower values suggesting parasympathetic predominance. However, this interpretation has been increasingly challenged. Because LF power does not exclusively represent sympathetic activity, the LF/HF ratio is now considered an oversimplified and potentially misleading marker of autonomic balance (Billman, 2013; Hayano and Yuda, 2019). In practice, spectral indices are typically calculated over short, spectral indices are generally computed from short, stationary segments (commonly 5-min epochs) that are averaged across the full recording period to improve reliability. Furthermore, numerical values, power spectral density (PSD) plots are frequently inspected visually, as well-organized spectral peaks and band distributions can be readily evaluated through graphical representation (Catai et al., 2020; Task Force of the European Society of Cardiology and the North American Society of Pacing and Electrophysiology, 1996). Consequently, spectral indices should be interpreted cautiously and ideally integrated with time-domain and nonlinear measures for comprehensive evaluation.

#### 2.1.3 Nonlinear analyses

Because cardiovascular control mechanisms are inherently complex and nonlinear, traditional linear metrics may not fully capture the dynamic structure of HRV (Task Force of the European Society of Cardiology and the North American Society of Pacing and Electrophysiology, 1996). Accordingly, nonlinear approaches including Poincaré plot analysis, entropy-based indices (approximate, sample, and multiscale entropy), symbolic dynamics, and detrended fluctuation analysis (DFA) have been developed to quantify the irregularity and self-similar complexity of heart rate behavior (Voss et al., 2009).

Among these, the Poincaré plot provides an intuitive geometric representation of beat-to-beat dynamics by plotting each R–R interval against the next. The short-axis (SD1) reflects short-term variability predominantly mediated by vagal activity, whereas the long-axis (SD2) represents longer-term modulation involving both autonomic branches. An elongated plot (high SD2/SD1 ratio) may indicate reduced autonomic flexibility or sympathetic predominance, while a compact, rounded pattern reflects strong adaptability (Matusik et al., 2023). Poincaré analysis thus offers both visual and quantitative insight into rhythm organization, even within nominally sinus rhythm, revealing patterns associated with sleep-disordered breathing, impaired baroreflex control, autonomic dysregulation, or cardiometabolic risk.

Entropy- and fractal-based measures further quantify how HRV complexity diminishes under pathological conditions characterized by impaired autonomic responsiveness or disrupted feedback control (Reyes-Lagos et al., 2019; Spellenberg et al., 2020). Collectively, nonlinear metrics complement time- and frequency-domain analyses by capturing the adaptive, self-organizing nature of cardiovascular regulation and offering sensitive indicators of autonomic balance and physiological resilience.

See Table 1 for nomenclature and definitions of common HRV indices.

**Table 1:** Common heart rate variability (HRV) indices, their measurement domains, definitions, and physiological significance.

HRV index	Type	Definition	Physiological significance
HR	Time-domain	Average heart rate during recording (beats/min)	Provides baseline cardiac activity information
SDRR	Time-domain	Standard deviation of all RR intervals, including abnormal/ectopic beats	Reflects total RR variability, non-autonomic factor can inflate values
SDNN	Time-domain	Standard deviation of normal-to-normal (NN) intervals	Reflects overall HRV and long-term autonomic regulation
RMSSD	Time-domain	Root mean square of successive differences between adjacent NN intervals	Reflects short-term parasympathetic (vagal) activity
pNN50	Time-domain	Percentage of NN intervals differing by >50 ms	Indicates parasympathetic modulation
VLF	Frequency-domain	Very low-frequency power (<0.04 Hz)	Associated with thermoregulation and long-term regulatory processes
LF	Frequency-domain	Low-frequency power (0.04–0.15 Hz)	Represents both sympathetic and vagal influences; interpretation is controversial
HF	Frequency-domain	High-frequency power (0.15–0.40 Hz)	Reflects parasympathetic (vagal) activity, particularly respiratory-related variability
LF/HF Ratio	Frequency-domain	Ratio of LF to HF power	Often used as an index of sympathovagal balance, though its physiological interpretation is controversial due to LF
LFnu	Frequency-domain (normalized)	Low-frequency power in normalized units: LF/(LF + HF) × 100	Indicates the relative contribution of LF to total variability; used to assess sympathovagal modulation (interpretation debated due to LF)
HFnu	Frequency-domain (normalized)	High-frequency power in normalized units: HF/(LF + HF) × 100	Indicates the relative contribution of HF power; reflects parasympathetic modulation
Total Power (TP)	Frequency-domain	Total variance in HRV signal, including VLF, LF, and HF components	Reflects overall autonomic nervous system activity
SD1	Nonlinear (Poincaré)	Standard deviation of points perpendicular to the line of identity	Reflects short-term HRV and parasympathetic activity
SD2	Nonlinear (Poincaré)	Standard deviation of points along the line of identity	Reflects overall HRV and long-term variability
Approximate Entropy (ApEn)	Nonlinear	Quantifies regularity and complexity of HRV time series	Lower values indicate reduced complexity, linked to aging and disease
Sample Entropy (SampEn)	Nonlinear	Measures unpredictability of HRV fluctuations	Lower values suggest reduced adaptability and autonomic dysfunction
DFA (α1)	Nonlinear (Fractals)	Short-term fractal scaling exponent from detrended fluctuation analysis	Reflects fractal-like HRV dynamics and autonomic regulation

Descriptions adapted from established HRV references (Schafer and Vagedes, 2013; Voss et al., 2009; Billman, 2013; Ernst, 2017; Shaffer and Ginsberg, 2017; Task Force of the European Society of Cardiology and the North American Society of Pacing and Electrophysiology, 1996).

Despite extensive research, consensus has yet to be reached regarding optimal recording duration, measurement conditions, or criteria for meaningful HRV assessment, underscoring the need for standardized data quality and interpretation guidelines (Shaffer and Ginsberg, 2017). RV research quality also depends critically on the characteristics of the recorded signal and the criteria used to define acceptable data. Studies that are similar in scientific intent can differ markedly in how HRV is collected, for example, in the population studied, the ECG channel used, or the duration of the recording. High-fidelity HRV assessment requires that data be derived from normal sinus beats with consistent R-wave peak detection across the entire recording. Uneven beat detection, signal noise, or inconsistencies across leads can introduce artificial variability, although some of these issues may be mitigated by adjusting scanning parameters. Clear reporting of participant characteristics, signal type, recording duration, and data-quality criteria is therefore essential for ensuring comparability across studies and enabling meaningful interpretation of HRV findings.

### 2.2 The physiological basis of HRV

HRV arises from the continuous interplay between the heart and the autonomic nervous system, mediated by biochemical, electrical, and reflexive feedback mechanisms. These interactions enable rapid adjustments to internal and external demands, allowing cardiovascular function to remain flexible and resilient. Thus, HRV reflects the integrity of multi-level physiological communication across the heart, brain, and vasculature.

### 2.3 HRV in aging and health outcomes

HRV is a valuable predictive marker for cardiovascular morbidity and mortality, overall health, and wellbeing (Kleiger et al., 1987; Thayer et al., 2010; Hillebrand et al., 2013; Zhou et al., 2016; Sen and McGill, 2018; Jarczok et al., 2022), as well as for the progression of aging (Almeida-Santos et al., 2016; Young and Benton, 2018; Manser et al., 2021; Calderon-Juarez et al., 2023). Its age-related decline largely reflects natural aging rather than being driven solely by cardiometabolic conditions or medication use (Jandackova et al., 2016; Jarczok et al., 2022). Because HRV reflects autonomic adaptability, it serves as a key tool for assessing resilience to stressors, disease risk, and intervention efficacy. Importantly, HRV is modifiable through pharmacological and non-pharmacological strategies, including lifestyle interventions, making it a promising target for improving cardiovascular outcomes and mitigating aging-related risks (Thayer et al., 2010).

Over time, HRV has emerged as a unifying biomarker linking autonomic regulation with systemic aging. Its associations with cardiovascular, metabolic, and psychological outcomes underscore its broad relevance (An et al., 2020; Souza et al., 2021). By providing a comprehensive lens into aging biology, HRV informs strategies to enhance longevity and healthspan. The following sections explore cardiovascular and neurological aging, examining mechanisms underlying HRV and its modulation through evidence-based interventions.

Reliable HRV assessment requires careful attention to data quality and completeness. Studies using 24-h Holter monitoring emphasize stringent inclusion criteria, artifact screening, and segment-quality standards to minimize the impact of arrhythmias and non-sinus intervals (Stein et al., 2009; Stein et al., 2012). Defining adequate recording duration and analytic thresholds is essential for comparability across studies and clinical applications.

## 3 HRV in cardiovascular and neurological aging

The heartbeat is driven by cardiac automaticity, where electrical impulses are spontaneously generated through the orchestrated opening and closing of ion channels across pacemaker cell membranes in the atrial sinus node. These oscillations in membrane potential propagate through the cardiac conduction system, creating rhythmic contractions of the myocardium to pump blood throughout the body (Zhang, 2021). Heart rate regulation is a highly complex and dynamic process involving multiple mechanisms, and responsive to physiological and pathological changes within and beyond the cardiovascular system.

A key regulatory mechanism is the baroreceptor reflex, initiated by mechanoreceptors in the carotid arteries and aortic arch. These receptors detect blood pressure fluctuations and relay signals to the central autonomic network, which adjusts pacemaker activity, cardiac contractility, and vascular tone to maintain homeostasis and stable perfusion to vital organs (Zhang and Wang, 2001; Zhang, 2006). With aging, baroreflex sensitivity declines due to reduced autonomic responsiveness, sympathetic-parasympathetic imbalance, and diminished buffering (Jones et al., 2003; De Meersman and Stein, 2007; Monahan, 2007). Experimental interruption of baroreflex input in animals confirms its essential role in maintaining HRV and cardiovascular complexity (Silva et al., 2015).

Age-related HRV decline results from three interconnected factors: autonomic dysregulation, intrinsic cardiac changes, and a pro-inflammatory, pro-oxidative milieu (Umetani et al., 1998; De Meersman and Stein, 2007; Monahan, 2007; Soares-Miranda et al., 2014; Williams et al., 2019). First, the ANS exhibits a progressive loss of flexibility with age, including reduced vagal modulation, attenuated baroreflex gain, and heightened sympathetic tone (Huang et al., 2007; de Matos et al., 2025). Neuroanatomical and neurochemical changes, such as impaired norepinephrine reuptake and decreased acetylcholine release, further disrupt cardiac autonomic signaling (Monahan, 2007; Kaye and Esler, 2008; Porta et al., 2014; Voss et al., 2015; Milan-Mattos et al., 2018) impaired pacemaker cell coupling, and reduced β-adrenergic receptor sensitivity, contribute to a decline in sinus node responsiveness (Lakatta and Levy, 2003; Choi et al., 2022; Arakaki et al., 2023). Third, the systemic pro-inflammatory and pro-oxidative state of aging (“inflammaging”) amplifies autonomic imbalance and myocardial remodeling through cytokine-mediated oxidative stress and mitochondrial dysfunction (Liberale et al., 2022; Fulop et al., 2019; Baechle et al., 2023). These changes contribute not only to reduced HRV magnitude but also to deterioration in signal quality. This qualitative change is captured by advanced measures like Heart Rate Fragmentation (HRF), a recently recognized phenomenon reflecting erratic short-term oscillations unrelated to smooth autonomic modulation. The prominence of HRF increases with advancing age and structural heart disease (Costa et al., 2017; Arakaki et al., 2023), and its presence may confound the interpretation of traditional, linear HRV metrics.

Numerous studies have documented an overall decline in HRV among healthy individuals with age (Pikkujamsa et al., 1999; Almeida-Santos et al., 2016; Hernandez-Vicente et al., 2020; Calderon-Juarez et al., 2023). Large-scale wearable-device studies, including one analyzing HRV from over 8 million Fitbit users in a single day, further reinforce the association between HRV, aging, wellbeing, and mortality risk (Natarajan et al., 2020).

The prognostic value of HRV for cardiovascular health was first recognized decades ago. A seminal study of 808 post-myocardial infarction (MI) survivors identified the standard deviation of all normal RR intervals (SDNN) as a critical HRV metric. Patients with SDNN values below 50 ms exhibited a 5.3-fold higher mortality risk compared to those with values exceeding 100 ms, even after adjusting for clinical and demographic factors (Kleiger et al., 1987). This study introduced HRV as a tool for cardiac risk stratification and stimulated decades of research into autonomic markers of cardiovascular prognosis. Importantly, these findings reflected clinical practice of the early 1980s, prior to widespread use of beta-blockers, angiotensin-converting enzyme inhibitors (ACEI), statins, and interventional procedures, so the specific SDNN thresholds no longer directly apply to contemporary post-MI populations. Despite these historical limitations, HRV remains a powerful, noninvasive biomarker of autonomic and cardiovascular integrity, providing valuable insight into physiological systems that can be modulated to improve cardiac outcomes. Subsequent studies have refined HRV-based risk models using modern cohorts and updated treatment standards; for example, an SDNN below 75 ms remained independently predictive of cardiac mortality (Stein, 2002; Barthel et al., 2003). HRV cut points and predictive strength can vary with recording duration, patient characteristics, and analytic criteria, underscoring the need for standardized measurement conditions in contemporary research.

Meta-analyses further demonstrate the predictive value of HRV. A 28-cohort study of cardiovascular patients found reduced HRV associated with a 112% increase in all-cause mortality (hazard ratio: 2.12, 95% confidence interval: 1.64–2.75) and a 46% increase in cardiovascular events (hazard ratio: 1.46, 95% confidence interval: 1.19–1.77) (Fang et al., 2020). Another meta-analysis of 32 studies with 38,008 participants confirmed that lower HRV correlates with higher all-cause and cardiac mortality, independent of age, sex, or study design (Jarczok et al., 2022).

Neurological aging is closely linked to HRV (Arakaki et al., 2023). The ANS, particularly parasympathetic regulation, modulates cardiac performance, while the cardiovascular system supports the brain through nutrient delivery, metabolite clearance, and optimal perfusion. HRV reflects this bidirectional interplay, providing insight into neurodegenerative conditions (Arakaki et al., 2023).

One prominent mechanism contributing to both cardiovascular and neurological aging is chronic inflammation, a process that becomes increasingly pronounced with advancing age (Liberale et al., 2022; Baechle et al., 2023; Li et al., 2023). The phenomenon, referred to as “inflammaging” (Fulop et al., 2019). Notably, studies have demonstrated a negative association between HRV and inflammatory markers, with SDNN and HF identified as particularly sensitive indices of this relationship (Williams et al., 2019). Age-related reductions in parasympathetic nerve activity, mediated via the cholinergic anti-inflammatory pathway, and concurrent increases in sympathetic nerve activity are strongly associated with inflammaging. This inflammatory process may sustain autonomic imbalance by promoting sympathetic dominance, thereby creating a self-reinforcing cycle (Giunta et al., 2024; Olivieri et al., 2024). The resultant cardiovascular and neuroinflammatory responses accelerate systemic aging and contribute to the pathogenesis of numerous age-related diseases (Shabab et al., 2017; Ferrucci and Fabbri, 2018; de Almeida et al., 2020). This reciprocal relationship positions HRV as both a biomarker and potential target for interventions to mitigate aging-related pathophysiology.

The general decline in HRV with age reflects a shift from complexity toward simplicity, consistent with the concept of entropy (Takahashi et al., 2012), a fundamental principle in physics. Nonlinear HRV indices—approximate entropy (ApEn), sample entropy (SampEn), detrended fluctuation analysis (DFA α1 and α2), and Poincaré plot parameters (SD1 and SD2) have been developed to capture the complex and chaotic dynamics of autonomic regulation that are not discernible through conventional time- or frequency-domain analyses (Voss et al., 2009). These measures quantify irregularity, self-similarity, and long-range correlations in RR intervals, providing generally indicate a loss of physiological complexity. Reduced nonlinear metrics with aging generally indicate a loss of physiological complexity; however, in older individuals, apparent HRV or entropy measures may be influenced by subtle rhythm disturbances or fragmented sinus activity, which can mimic high variability without reflecting true autonomic flexibility. This is particularly relevant in short-term recordings (e.g., 5-min epochs), where the ECG may appear as normal sinus rhythm despite underlying irregularities that Poincaré plots can reveal. Mechanisms underlying age-related changes likely involve alterations in neurotransmitter release, receptor sensitivity, and modulatory processes such as respiration (McCraty and Shaffer, 2015; He, 2020; Tiwari et al., 2021; Arakaki et al., 2023; Tegegne et al., 2023).

HRV’s dual relevance to cardiovascular and neurological systems underscores its role as a unifying marker of systemic aging. Its age-related decline reflects interconnected vulnerabilities of the heart and brain. Preserving or enhancing HRV through lifestyle, nutritional, pharmacological, or technological approaches may mitigate adverse aging effects, advancing strategies to promote longevity and healthspan.

## 4 Dietary influences on HRV: current evidence and mechanisms of action

Research on dietary influences on heart rate variability (HRV) has expanded substantially, yet the evidence remains heterogeneous and fragmented. Interventions have been studied in both healthy individuals and clinical populations with conditions such as metabolic syndrome or heart failure that impair HRV. This section focuses on four illustrative interventions—omega-3 fatty acids, vitamin B12, caloric restriction, and probiotics chosen because they are supported by a sufficient number of human studies and exhibit mechanistically explainable links to autonomic regulation. This selection does not imply that other dietary factors are unimportant; rather, it highlights examples with both experimental support and biologically plausible mechanisms, providing a framework for interpreting emerging findings. By concentrating on these interventions, the section emphasizes current insights while acknowledging knowledge gaps and the need for systematic, comparative studies to further clarify dietary effects on HRV.

### 4.1 Omega-3 and HRV

Over the past two decades, numerous studies have investigated how omega-3 polyunsaturated fatty acids (PUFAs) influence heart rate variability (HRV). Reviews and meta-analyses have synthesized this literature, highlighting omega-3’s potential to enhance HRV and reduce arrhythmic events and sudden cardiac death (SCD).

A 2011 review examined modulations studies published between 1996 and 2011, including a diverse range of participants, from infants to adults and from healthy individuals to those with conditions such as ischemic heart disease, diabetes mellitus, and chronic renal failure (Christensen, 2011). Among the 20 studies reviewed, 8 reported a beneficial effect of omega-3 on HRV, 7 demonstrated benefits in subgroup analyses, and 5 found no effect. The findings might appear to reflect the heterogeneity across studies. Key factors include differences in omega-3 dosages (0.9–6.6 g/day), modulations durations (4 weeks to 6 months), sample sizes (*n* = 10–102), health statuses, and HRV assessment methodologies. Nonetheless, the overall evidence suggested that omega-3 supplementation may enhance HRV and partially explain its association with lower arrhythmic risk and SCD incidence (Christensen, 2011).

A 2013 meta-analysis further evaluated 18 comparisons from 15 randomized controlled trials, including 692 participants (349 in the omega-3 group and 343 in the control group) with a median age of 53 years (range: 38–77 years) (Xin et al., 2013). The studies included diverse populations: three involved generally healthy individuals, one focused on elderly nursing home residents, four included obese or overweight individuals, and nine involved patients with one or more chronic conditions such as dyslipidemia, coronary artery disease, chronic renal failure, idiopathic dilated cardiomyopathy, and epilepsy. The median daily omega-3 dosage was 1,680 mg of EPA and DHA (range: 640–5,900 mg/day, with EPA-to-DHA ratios varying from 0.2 to 1.5). The median duration of supplementation was 12 weeks (range: 6–24 weeks) (Xin et al., 2013). Time-domain indices (SDNN, RMSSD) were not significantly altered, but frequency-domain analysis showed a significant increase in HF power, a marker of vagal activity. Using a random-effects model, a standardized mean difference (SMD) for HF was 0.30 (*p* = 0.005), indicating a favorable vagal effect (Xin et al., 2013).

Beyond pooled analyses, several contexts highlight the impact of omega-3 intake on HRV: 1. *Pregnancy and Early Life*: Professional organizations recommend omega-3 intake during pregnancy and lactation for maternal and fetal benefits. Supplementation improves HRV in mothers, fetuses, and neonates (Gustafson et al., 2013; Christifano et al., 2023), indicating enhanced vagal regulation and potentially greater neonatal survival (Oliveira et al., 2019). From the Developmental Origins of Health and Disease (DOHaD) perspective, early-life nutrition, particularly during the first 1,000 days, may exert epigenetic effects (Agosti et al., 2017; Hoffman et al., 2017). Omega-3 supplementation during pregnancy has been shown to influence developmental programming mechanisms (Amatruda et al., 2019; Gonzalez-Becerra et al., 2019), though persistence into later life remains to be clarified. 2. *Chronic Kidney Disease (CKD)*: Sympathetic overactivity or imbalance between sympathetic and vagal tone, is common in CKD (Augustyniak et al., 2002; Tuncel et al., 2002; Grassi et al., 2021). Omega-3 supplementation improves HRV in CKD patients, including renal transplant recipients and those undergoing dialysis (Rantanen et al., 2018; Lilleberg et al., 2019; Pinto et al., 2021). This effect may be mediated by enhanced vagal activity or suppression of sympathetic activity (Singer et al., 2008; Zhang, 2021), and appears more pronounced in individuals with higher sympathetic tone or low vagal tone. 3. *Environmental Pollution*: Exposure to air pollutants, such as particulate matter (PM2.5), nitrogen dioxide (NO2) and ozone (O3) is a recognized environmental stressor that reduces HRV (Dietrich et al., 2008; Zhang et al., 2015; Zong et al., 2022). Omega-3 supplementation mitigates these pollution-induced reductions in HRV (Zhang et al., 2015; Chen et al., 2021; Chen et al., 2022), highlighting its potential as an intervention to counteract environmental harms.

Several large-scale trials involving omega-3 interventions have collected ECG data at baseline and during follow-ups (e.g., REDUCE-IT trial) but focused primarily on reporting primary or secondary endpoints, such as cardiovascular death, non-fatal myocardial infarction, non-fatal stroke, coronary revascularization, or unstable angina (Bhatt et al., 2019). Revisiting and analyzing HRV data could provide valuable insights into the effects of omega-3 supplementation on heart rate and rhythm (Zhang et al., 2024b). Moreover, such analyses could help establish whether changes in HRV are associated with survival outcomes, offering a predictive framework to determine how HRV responses to omega-3 may differentiate likely survivors from non-survivors (Zhang et al., 2024b).

The improvement of HRV by omega-3 can be attributed to several mechanisms: 1. *Sympathetic and Parasympathetic Balance*: Omega-3 improves the balance between sympathetic and parasympathetic activity by augmenting vagal tone and inhibiting sympathetic outflow, as previously discussed (Zhang, 2021). 2. *Baroreflex Sensitization*: While this effect of omega-3 is not observed in healthy young individuals with normal baroreflex function (Macartney et al., 2022), omega-3 supplementation has been shown to potentiate baroreflex sensitivity and enhance HRV in heart failure patients with blunted baroreflex function (Radaelli et al., 2006). 3. *Specialized Pro-Resolving Mediators (SPMs)*: Metabolites of omega-3, including protectins (PD), maresins (MaR), and resolvins D (RvD) and E (RvE), act as specialized pro-resolving mediators with anti-inflammatory and antioxidant properties (Giacobbe et al., 2020; Zhang et al., 2020). 4. *Structural Lipid Roles*: Omega-3 fatty acids, particularly DHA, are critical structural components of cellular membranes. Changes in omega-3 content can influence membrane fluidity, cell signaling, and the production of lipid mediators (Calder, 2010). Together, these findings support omega-3 fatty acids as modulators of autonomic balance, inflammation, and cellular signaling, thereby improving HRV and potentially reducing cardiovascular risk.

### 4.2 Vitamin B12 (cyanocobalamin) and HRV

Vitamin B12 is an essential micronutrient involved in neural development, myelination, and higher-order brain functions such as mood, memory, and cognition (Kobe et al., 2016; Allen et al., 2018; Calderon-Ospina and Nava-Mesa, 2020; Mathew et al., 2024). Most human evidence linking vitamin B12 to autonomic regulation and HRV comes from deficiency states, where impaired methylation, demyelination, oxidative stress, and endothelial dysfunction collectively compromise neural and cardiovascular control (Beitzke et al., 2002; Allen et al., 2018). A few investigations also show HRV improvements following supplementation, although data in individuals without deficiency remain limited.

In a 2000 study, patients with vitamin B12 deficiency and megaloblastic anemia (*n* = 18) exhibited significantly lower LF, LFNU, HF, HFNU, and LF:HF ratio compared with matched healthy controls (*n* = 15). All HRV indices correlated positively with serum vitamin B12 levels (*p* < 0.001) and negatively with the duration of deficiency (ranging from 6 months to 4 years). After 3 months of supplementation, hemoglobin levels were comparable between the two groups (14.1 ± 1.6 mg/dL vs. 14.5 ± 0.9 mg/dL), resulting HRV parameters largely normalized, despite the study not reporting exact dosage (Aytemir et al., 2000).

Similarly, a 2012 study found that elderly adults with vitamin B12 deficiency (mean 87.4 ± 33.0 pmol/L) exhibited significantly lower LF power compared with B12-sufficient older adults. Daily supplementation with 100 μg vitamin B12 increased LF power and total HRV power in deficient participants (Sucharita et al., 2012).

Air pollution, particularly PM2.5, is known to reduce HRV, with the most pronounced decreases observed in individuals with low intake of methyl donors such as vitamins B6, B12, and methionine Conversely, higher intake of these nutrients mitigates PM2.5-induced reductions in HRV (Zhang et al., 2015). In a controlled crossover intervention study, 10 healthy adults were exposed to 2 h of clean air, PM2.5, and PM2.5 with B-vitamin supplementation. PM2.5 exposure reduced LF power by 57.5% (95% confidence interval: 2.5, 81.5%; *p* = 0.04), but supplementation with folic acid (2.5 mg/day), vitamin B6 (50 mg/day), and vitamin B12 (1 mg/day) significantly attenuated this effect. However, the study’s small sample size limited its ability to detect the effects on other HRV indices (Zhong et al., 2017). While reduced HRV due to deficiency can be restored, Whether B12 supplementation enhances HRV in non-deficient individuals remains unclear.

Mechanistically, B12 deficiency disrupts autonomic regulation primarily through compromised myelin integrity and increased neuroinflammation. Vitamin B12 is a cofactor for methionine synthase, which generates S-adenosylmethionine (SAM), a universal methyl donor required for myelin maintenance. Deficiency lowers SAM, elevates methylmalonic acid (MMA) and homocysteine, and drives neuroinflammatory and oxidative pathways that impair autonomic signaling (Metz, 1992; Scalabrino, 2009). Elevated homocysteine increases oxidative stress and proinflammatory cytokines such as TNF-α, IL-1β, IL-6, MCP-1, and ICAM-1, affecting both cardiovascular regulation and HRV complexity (Djuric et al., 2018), potentially reducing HRV complexity (Williams et al., 2019). Because hyperhomocysteinemia is a hallmark of B12 deficiency (Hunt et al., 2014), vitamin B12 (often combined with folate and vitamin B6) is effective in lowering homocysteine (Sohouli et al., 2024) and may reverse deficiency-induced impairments in autonomic function (Ueno et al., 2022; Liu et al., 2023).

Overall, although some evidence supports HRV restoration in B12-deficient individuals, the potential for vitamin B12 to enhance HRV in the general, non-deficient population remains to be established.

### 4.3 Calorie restriction (CR) and HRV

Caloric restriction (CR), defined as a 20–40% reduction in calorie intake without malnutrition, is a widely recognized lifestyle intervention for promoting cardiovascular, metabolic, and neurological health (Zhang et al., 2024a). Its benefits extend to both humans and non-human primates, demonstrating its role in prolonging lifespan and improving overall health outcomes (Pifferi and Aujard, 2019). While CR is often addressed in the population of overweight and obesity that constitute a key driver of cardiovascular and metabolic risk in mid- and high-income economies, it is equally effective for individuals with normal body weight seeking enhanced healthspan and longevity (Zhang et al., 2024a).

CR offers a wide range of health benefits. It improves blood lipid profiles, blood pressure, insulin sensitivity, blood sugar, and supports weight management (Golbidi et al., 2017; Nicoll and Henein, 2018; Hofer et al., 2022). Additionally, CR reduces inflammation and oxidative stress by lowering pro-inflammatory cytokines and mitigating free radical damage (Golbidi et al., 2017; Kokten et al., 2021; Mehdi et al., 2021; Hofer et al., 2022), contributing to enhanced quality of life and extended lifespan. Mechanistically, CR induces a metabolic shift toward increased fatty acid oxidation and ketogenesis, leading to elevated circulating levels of β-hydroxybutyrate (3-hydroxybutyrate). This ketone body functions not only as an alternative energy substrate but also as a signaling metabolite that activates survival and longevity pathways, including SIRT1, AMPK, and FOXO, while inhibiting mTOR signaling (Rachakatla and Kalashikam, 2022). These molecular adaptations collectively promote mitochondrial efficiency, attenuate oxidative stress, and suppress pro-inflammatory gene expression, thereby enhancing autonomic stability and HRV (Yelisyeyeva et al., 2025).

Initiating CR early in adulthood may reduce the risk of age-related diseases such as cardiovascular, metabolic, and cognitive disorders. Data from the National Institute on Aging CALERIE randomized trial (CALERIE biobank) reveal that biological aging progresses at a rate of 0.71 ‘years’ per 12-month period in the control group but is decelerated to 0.11 ‘years’ per 12-month period in the CR group (Belsky et al., 2017; Zhang et al., 2024a). Consistent with the law of entropy, the lifespan-extending effects of CR may be attributed to reduced entropy generation (Semercioz-Oduncuoglu et al., 2023). Given its non-invasive, self-implementable, and affordable nature, CR represents a proven strategy for promoting health and longevity in both research and practical settings (Flanagan et al., 2020; Zhang et al., 2024a).

Emerging evidence links CR to enhancements in HRV, particularly through its capacity to balance sympathetic and parasympathetic nervous system activity (Facchini et al., 2003; Struven et al., 2021; Polito et al., 2022). Research on long-term fasting revealed that older participants achieved HRV outcomes comparable to much younger individuals or to hypertensive patients using atenolol, a medication that shifts autonomic control by decreasing sympathetic and enhancing parasympathetic activity (Stein et al., 2012). A study of 2 weeks of CR in hypertensive patients revealed notable changes in autonomic balance: high-frequency activity—a marker of parasympathetic function, increased during nighttime, while the low-frequency/high-frequency ratio, a marker of sympathetic dominance, decreased during daytime, suggesting an improvement in nocturnal autonomic regulation (Nakano et al., 2001). A 3-week body weight reduction program, combining an energy-restricted diet with high-intensity exercise, evaluated changes in HRV among severely obese, normotensive patients. HRV was measured using an 18-h Holter recording before and after the intervention. The program led to a 4.6% reduction in BMI (41.4 ± 4.6 to 39.5 ± 4.3 kg/m^2^, *p* < 0.0001), a significant decrease in heart rate (77.8 ± 8.6 to 73.6 ± 8.7 bpm, *p* = 0.0003), and improvements in both time and frequency domain metrics (e.g., SDRR: +16.1%; MSSD: +16.7%; pNN50: +31.8%; LF: +17.1%; HF: +18.2%) (Facchini et al., 2003). As discussed earlier, the physiological interpretation of LF power remains controversial, and the use of SDRR instead of SDNN may include noise from ectopic or abnormal beats. Nonetheless, consistent increases in other parasympathetically mediated indices, particularly MSSD, pNN50, and HF, support an enhancement of vagal modulation; however, the overall improvement in other HRV indices particularly MSSD, pNN50, and HF supports the hypothesis of enhanced parasympathetic activity. Although this review focuses on dietary approaches, it is worth noting that combining CR with exercise that is another active way of lifestyle modification known to improve HRV (Raffin et al., 2019), has been shown to produce even greater improvements in HRV (Facchini et al., 2003; de Jonge et al., 2010). For instance, CR has demonstrated its capacity to shift autonomic control favorably, with older participants achieving HRV outcomes comparable to younger individuals. A review of 33 studies on continuous CR and fasting in humans found that 23 reported significant cognitive improvements. Although the effects varied across cognitive domains, CR generally enhanced processing speed, working memory, and response inhibition, albeit with potential impairments in cognitive flexibility (O'Leary et al., 2025). Furthermore, there is also evidence that CR may cause structurally change in the brain, e.g., increased gray matter volume in inferior frontal gyrus and hippocampus (Prehn et al., 2017).

Overall, CR appears to consistently enhance HRV through reductions in inflammation and sympathetic activity, improved parasympathetic modulation, and metabolic reprogramming. Yet the optimal degree of restriction and the durability of HRV benefits remain open questions that require long-term and mechanistically oriented trials.

### 4.4 Probiotics and HRV

The gut–heart–brain axis concept positions the intestinal microbiota as a central regulator of autonomic balance and thus heart rate variability (HRV). Probiotics—live microorganisms that, when administered in adequate amounts, confer a health benefit, have gained attention as a targeted strategy to modulate the gut ecosystem and support cardiovascular/ autonomic health.

Lately, several studies have explored the field related to inflammatory markers and autonomic indices of gut microbiota and probiotic supplementation (Grant et al., 2025; Mörkl et al., 2025; Ravenda et al., 2025; Yu et al., 2025), of which a couple are worth noting. For instance, in women with hypertension, an 8-week multi-strain probiotic (including *Lactobacillus paracasei* LPC-37, *L. rhamnosus* HN001, *L. acidophilus* NCFM, *Bifidobacterium lactis* HN019) increased anti-inflammatory IL-10 and decreased INF-γ; INF-γ was positively correlated with heart rate and sympathetic HRV indices, and negatively with vagal indices (Maia et al., 2025). Although HRV change itself was not the main endpoint, these correlations support the pathway: certain probiotics—reduced inflammation—autonomic modulation. From an HRV-specific and microbiota-composition perspective: higher gut microbial diversity and abundance of certain genera (e.g., Lachnospiraceae incertae sedis) were associated with higher HRV parameters (e.g., SDNN) in a general-population study (Tsubokawa et al., 2022). Also, in healthy individuals, low vagally-mediated HRV was associated with altered gut microbiota profiles (Ravenda et al., 2025).

Mechanistically, dietary fiber fermentation (and probiotic-driven colonization) produces short-chain fatty acids (SCFAs) such as acetate, propionate and butyrate, which act as signaling molecules to reduce systemic inflammation (Fernández et al., 2016; Bruning et al., 2020), influence neuro-immuno-endocrine pathways and engage the vagus nerve.

Although dietary influences on HRV span a broad range of nutrients and eating patterns (Manzella et al., 2001; Almoznino-Sarafian et al., 2009; Park et al., 2009; Tunapong et al., 2018; Lopresti, 2020; Almeida et al., 2024), the four interventions highlighted here: omega-3 fatty acids, vitamin B12, caloric restriction, and probiotics, illustrate how nutrition can modulate autonomic regulation through overlapping yet distinct mechanisms. Omega-3 fatty acids enhance vagal activity and attenuate inflammation; vitamin B12 supports neuronal integrity and methylation; caloric restriction promotes metabolic flexibility, mitochondrial efficiency, and anti-inflammatory signaling; and probiotics modulate gut–brain communication via short-chain fatty acids and vagal pathways. Collectively, these mechanisms underscore HRV as a dynamic marker of autonomic adaptability and a potential target for nutritional interventions.

Importantly, the impact of diet on HRV is bidirectional: while certain nutrients or interventions may enhance autonomic function, others such as trans-fatty acids can impair HRV and resting heart rate in two longitudinal cohorts including both young and older adults (Soares-Miranda et al., 2012). Future research should focus on head-to-head comparisons, dose–response relationships, long-term effects, and mechanistic clarification, particularly in aging populations, to establish evidence-based dietary strategies for sustaining autonomic balance and reducing cardiometabolic risk.

## 5 Conclusions and perspective

Aging involves interconnected biological processes such as inflammaging, oxidative stress, impaired metabolic signaling, and autonomic decline that collectively contribute to reductions in heart rate variability (HRV). Yet these trajectories are modifiable rather than irreversible. Across diverse lines of evidence, several nutritional and lifestyle strategies have demonstrated the capacity to modulate autonomic function and support healthier HRV profiles.

Among these interventions, omega-3 polyunsaturated fatty acids remain the best characterized, with consistent evidence for enhancing vagal tone, reducing inflammation, and supporting membrane and mitochondrial function. Vitamin B12, essential for neurophysiological signaling and methylation capacity, may influence HRV through effects on autonomic nerve integrity, particularly in older adults where deficiency is prevalent. Caloric restriction via improvements in metabolic efficiency, mitochondrial resilience, and inflammatory tone has repeatedly been linked to enhanced autonomic balance and extended healthspan. Emerging probiotic and microbiota-targeted strategies also suggest gut–brain interactions as promising modulators of HRV and autonomic aging.

However, substantial knowledge gaps remain. Variability in study design, population characteristics, intervention duration, HRV analytic methods, and biomarker coverage limits comparability across existing trials. Future investigations would benefit from integrated designs that pair HRV endpoints with nutritional biomarkers, inflammatory and oxidative metrics, and microbiome or metabolomic profiling. Longitudinal and mechanistic studies are particularly needed to clarify causal pathways and individual responsiveness.

Overall, current evidence supports the view that HRV is not merely a marker of aging but a dynamic and modifiable physiological parameter. Progress in this field will advance precision-nutrition and lifestyle strategies aimed at preserving autonomic function, reducing cardiovascular vulnerability, and promoting healthy aging.
